# Figurative Language, Language Disorders, and Language(s) Evolution

**DOI:** 10.3389/fpsyg.2017.01713

**Published:** 2017-09-29

**Authors:** Antonio Benítez-Burraco

**Affiliations:** Department of Philology, University of Huelva, Huelva, Spain

**Keywords:** figurative language, language evolution, globularity, cross-modular thinking, domestication, cognitive disorders, socialization, evo-devo

As pointed out in the call-for-papers of this Research Topic, the ability to understand and make use of figurative language seems to be altered in most, if not all, cognitive disorders. In this Opinion paper, it is argued that putting the focus on the evolution of human cognition helps achieve a better understanding of the problems that clinical populations experience with figurative language, and particularly, of the nature of the involved neural devices and cognitive mechanisms. The ultimate reason is the deep link that exists between evolution and (abnormal) development, in the spirit of evo-devo theories.

Human cognition outscores in the ability to transcend the signature limits of core knowledge systems (Spelke, 2003; Wynn and Coolidge, 2011). Among other things, this ability enables to combine and unify conceptual units that belong to distinct domains. Most types of figurative language boil down to this ability. Paradigmatically, in metonyms and metaphors we refer to a target domain in terms of a source domain, because a real-word link (in metonymy) or a conceptual relationship (in metaphor) exist between both domains. Evolutionarily, this ability for cross-modular thinking seems to be a human innovation, that seemingly resulted from the brain rewiring linked to the emergence of our globular skull and brain. Accordingly, this anatomical shift reshaped the connections between several cortical and sub-cortical areas (particularly, the thalamus and the cerebellum) in the hominin brain and habilitated a new neuronal workspace, with more long-distant connections and more cognitive fluidity (see Boeckx and Benítez-Burraco, 2014 for details).

Interestingly, many candidate genes for the globularization of the human skull/brain and for our cognitive distinctiveness are also candidates for cognitive disorders entailing problems with figurative language, particularly, autism spectrum disorders (ASD) and schizophrenia (SZ) (see Murphy and Benítez-Burraco, 2016a, Table 2). The same happens with candidate genes for the patterns of brain activity underlying our mode of cognition, which result from phasal and cross-frequency coupling properties of neural oscillation that are species-specific (Murphy and Benítez-Burraco, 2017, Table 1). Overall, this suggests that the same genes that are mutated or altered in cognitive disorders entailing problems with figurative language were involved in the emergence of our mode of cognition. On the one hand, these cognitive disorders are thought to be human-specific conditions. On the other hand, the gene loci associated with them are enriched in genomic regions that have undergone positive selection in our species only, as the case of SZ nicely illustrates (Srinivasan et al., 2017). The human-specificity of these cognitive disorders and their high prevalence within modern populations is ultimately explained by the fact that the changes that brought about our mode of cognition pushed the primate cognition far away from the robust equilibrium achieved after millions of stabilizing selection and uncovered (or more properly, de-canalized) the cryptic variation existing in primates (see Gibson, 2009 for a characterization of complex disorders in humans as de-canalized conditions).

Nonetheless, this is not the full story. Most of the cognitive disorders entailing problems with figurative language also entail problems with core components of language, like phonology, semantics, or syntax, which, allegedly, concern to the literal meaning of utterances only. Again, ASD and SZ are paradigmatic examples (see Benítez-Burraco and Murphy, 2016; Murphy and Benítez-Burraco, 2016b for review). To a certain extent, this is not surprising, because as highlighted by cognitive linguistics, the boundaries between figurative and non-figurative language are fuzzy particularly, because core mechanisms involved in the former (like metaphorization) also account for nuclear aspects of the later (for instance, when we say *Next week we will meet George* to convey the thought that we are going to meet George in 7 days, this is not regarded as a figurative use of language, in spite that *next week* conceals the metaphor TIME is SPACE). Nonetheless, the ultimate reason could be that our ability to form cross-modular concepts (and thus, to metaphorize) can be conflated to *merge*, the core combinatorial operation in natural language, which combines elementary linguistic units to form more complex units (see Boeckx and Benítez-Burraco, 2014, for discussion). This ability is at the core of grammar, and ultimately, of our ability to learn and use languages (that is, our *language-readiness*).

Let us add a last chapter to this story. As noted by several evolutionary linguists, the human-specific ability for cross-modular thinking may have favored subsequent steps in the evolution of language, specifically, the increase of language complexity via grammaticalization (a process by which linguistic items start to convey grammatical meanings or reinforce their grammatical roles) and ultimately, the emergence of modern languages (see Benítez-Burraco, 2017, for discussion). Similarly to the use of figurative language, many aspects of grammaticalization boil down to the principles governing conversational exchange, particularly to pragmatic inferencing (Smith and Höfler, 2014). Importantly, the acquisition of pragmatic abilities has both a cognitive dimension (i.e., acquiring the capacity for cross-modular thinking, surely, but also the ability to understand others' intentions) and a social dimension (i.e., learning about social relations for mastering politeness or learning to do things with words for correctly performing speech acts) (Zufferey, 2015). Notice that people suffering from cognitive disorders entailing problems with figurative language (and as noted, deficits in core aspects of language) also experience problems with social interactions. ASD is a classic example. Although the problems that people with ASD have with figurative language have been explained in terms of either a cognitive deficit affecting the Theory of Mind (Happé, 1995), or a language deficit *per se*, particularly, in semantic competence (Norbury, 2004), we cannot ignore that mastering both abilities needs a rich social environment (see Syal and Finlay, 2011; Burnside et al., 2017, and references therein). Following our line of evidence, our hypothesis is that these disparate deficits (problems with figurative language, with non-figurative language, and with socialization) result from the impairment of a common underlying biological mechanism that was remodeled during our recent evolutionary history.

Ongoing research on language evolution also suggests that modern languages, endowed with the full impedimenta which is familiar to linguists, only emerged when human beings, being cognitively modern, succeeded as well in constructing the socio-cultural niche that, among many other things, allowed language complexity to increase through a cultural process. In brief, this niche provides the child with an extended socialization window that enables him to interact with other people more safely, more regularly, and for a longer time. Specifically, several researchers have argued that this special niche resulted in part from the self-domestication of the human species (see Hare and Tomasello, 2005; Thomas, 2014 among others, for details).

Intriguingly, most cognitive disorders in which the use of figurative language is impaired (and which show problems with structural components of language and altered socialization patterns) exhibit an abnormal presentation of “domesticated” traits in humans, from brain structure to behavioral features (see Benítez-Burraco et al., 2016a on ASD and Benítez-Burraco et al., 2017 on SZ). Self-domestication of our species has been explained in terms of an adaptation to the human-made environment, or as the result of selection against aggression (see Thomas, 2014, for details). However, we have recently found that many candidate genes for the domestication of mammals are also candidates (or interact with candidates) for the globularization of the human skull/brain (see Benítez-Burraco et al., 2016b for details). This suggests that human self-domestication might be, to some extent, a by-product of the same changes that brought about our species-specific mode of cognition, encompassing both complex language and the ability to process figurative language (Figure 1A).

**Figure 1:**
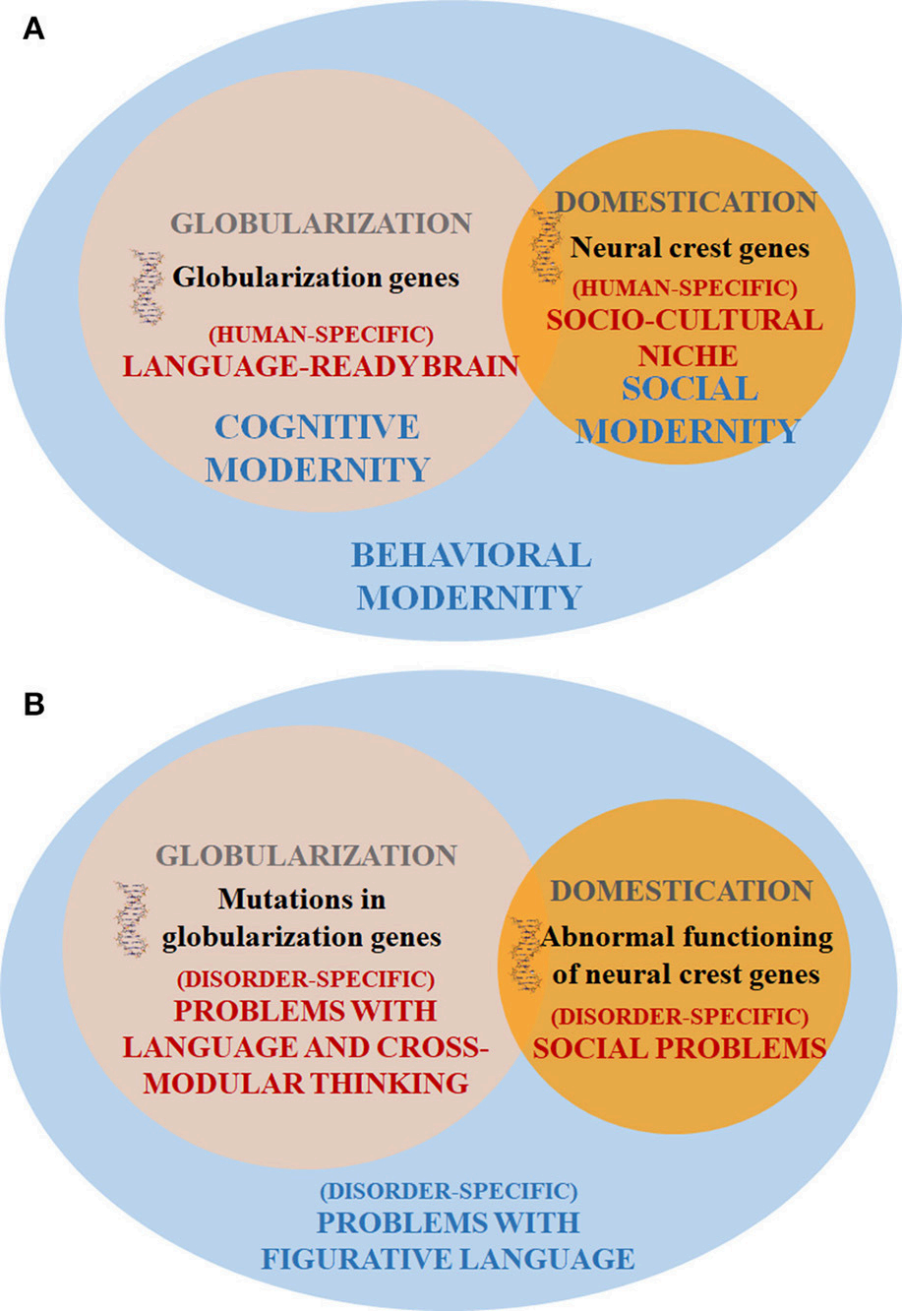
Outline of an Evo-Devo approach to problems with figurative language in populations with cognitive disorders. **(A)** Outline of the evolutionary processes responsible for the emergence of our language-readiness and of complex languages. **(B)** Outline of the three aspects contributing to the problems with figurative language experienced by people suffering from cognitive disorders.

It is not then surprising that these three facets of the human phenotype (i.e., non-figurative language, figurative language, and social behavior) are found altered in most, if not all, cognitive disorders. Because the evolution of the three of them is tightly interwoven, mutations in any of the candidate genes for the process will result in problems in the three domains (Figure 1B). Importantly, as noted by Wilkins et al. (2014), many of the genes related to the domestication of mammals are involved in the development and function of the neural crest. Although this possibility has to be experimentally tested, we expect this finding to provide with a single biological explanation of the co-occurrence of linguistic, cognitive, and behavioral deficits in disorders entailing problems with figurative language.

In sum, we anticipate that the study of the evolutionary trajectory of human language illuminates the problems that people with cognitive disorders usually experience with figurative language. Specifically, we expect that this line of research helps accommodate many of the hypotheses, sometimes disparate, that have been formulated to account for such problems. As with other aspects of language processing, development, and evolution, we advocate for an approach to this issue that heavily focuses on the oscillatory signature of the brain. For instance, language deficits in ASD can be successfully tracked to an abnormal oscillatory behavior of the autistic brain (Benítez-Burraco and Murphy, 2016), that can be related, in turn, to the inferred changes in the oscillatory dynamics of the hominin brain during recent human evolution (Murphy and Benítez-Burraco, 2017). Ultimately, we support an evo-devo approach to this problem, aimed to disentangle how the specific patterns of cortical inhibition and long-distance connections across the brain that underlie our species-specific ability to form and exploit cross-modular concepts develop in the child, evolved in the species, and are impaired in people with cognitive disorders.

## Author contributions

The author confirms being the sole contributor of this work and approved it for publication.

### Conflict of interest statement

The author declares that the research was conducted in the absence of any commercial or financial relationships that could be construed as a potential conflict of interest.
